# Parenting Practices and Adolescent Effortful Control: *MAOA* T941G Gene Polymorphism as a Moderator

**DOI:** 10.3389/fpsyg.2020.00060

**Published:** 2020-02-18

**Authors:** Bao Zhao, Yanmiao Cao, Liang Zhang, Wenxin Zhang

**Affiliations:** Department of Psychology, Shandong Normal University, Jinan, China

**Keywords:** effortful control, monoamine oxidase A, parenting practices, gene–environment interaction, adolescent

## Abstract

Effortful control (EC) plays a crucial role in psychopathology disorders. Emerging studies have paid attention to the effects of G × E interaction on EC. The present study investigated interactions between monoamine oxidase A (*MAOA*) T941G polymorphism with parenting practices on EC in a sample of 1,531 Chinese adolescents. The adolescents completed the Early Adolescent Temperament Questionnaire–Revised (EATQ–R) EC scale and the Parenting Style Index provided during the study to assess EC and parenting practices, respectively. *MAOA* T941G polymorphism exerted no effect on adolescent EC; however, results revealed that the *MAOA* gene interacted with parental acceptance/involvement in their associations with EC among boys. Specifically, although increased levels of parental acceptance/involvement benefited all adolescents, boys with G alleles of the *MAOA* gene exhibited higher sensitivity to parental acceptance/involvement, compared with T carriers; this interaction was not significant among girls. This study is the first to identify *MAOA* × parenting interaction on adolescent EC, further contributing to the literature in *MAOA* gene–EC.

## Introduction

Effortful control, the self-regulatory aspect of temperament, is defined as the ability to inhibit a dominant response to perform a subdominant response, detect errors, and engage in planning (Ellis and Rothbart, 2001; Eisenberger et al., 2005; Rothbart and Bates, 2006). This ability is associated with emotional and behavioral regulation and includes effortful modulation of attention (focusing and directing attention), inhibition control (suppressing dominant responses), and activation control (performing an action when intrinsic motivation is lacking) (Rothbart and Posner, 2005). EC plays a crucial role in the development of psychopathology, particularly externalizing (Doan et al., 2012) and internalizing problems (Rothbart et al., 2003). Deficits in EC can have severe consequences; thus, its antecedents need to be examined.

Effortful control, an aspect of temperament, is associated with genetic heritability (Gagne et al., 2011) and is a relatively stable, upstream aspect of human behavior. However, evidence also indicates that temperamental experience and expression are shaped by context and experience (Rothbart and Bates, 2006). As a key aspect of such experience in childhood, parenting has been proved to play an important role in the development of EC (Kiff et al., 2011; Stacey et al., 2016). A large literature showed that the robust, replicated findings of beneficial effects of positive environments for EC draw from parenting (Karreman et al., 2006). Compared with children exposed to less maternal empathic, accepting, and supportive parenting behaviors, those who received more positive parenting exhibited better self-regulation (Eisenberg et al., 1998, 2010). Maternal responsiveness has also been associated with self-regulation in adolescents (Doan et al., 2012). Thus, positive parental behaviors exhibit potential as a promotive factor for EC development.

Emerging studies have examined the genetic factors underlying EC, next to environmental factors. Evidence from twin studies has estimated the heritability of EC to reach 79% (Lemery-Chalfant et al., 2008). Neural and neurochemical studies have suggested that genes implicated in monoamine function are important candidate genes for EC. For instance, dopamine regulates activity in the prefrontal cortex and has been linked to self-control in animal studies (Rodriguiz et al., 2004). Animal and human studies have found that serotonin underlies behavioral inhibition. Serotonin has also been associated with neural regions belonging to the executive attention network involved in self-regulation (Posner et al., 2007). Matrenza et al. (2004) have indicated that experimental depletion of serotonin and catecholamines impair sustained attention, a primary component of EC. Thus, genetic variants implicated in monoamine (e.g., dopamine and serotonin) function are potential genes for EC.

The *MAOA* gene has been proposed as an important candidate gene for EC because it contributes to the catabolism of monoamine neurotransmitters, including dopamine, serotonin, and norepinephrine (Shih et al., 1999). The *MAOA* gene is located on the X chromosome at Xp.11.3–Xp11.4. Most studies on the *MAOA* gene have thus far focused on the variable number tandem repeat (VNTR) in the upstream promoter region of the *MAOA* gene (Sabol et al., 1998; Buckholtz and Meyer-Lindenberg, 2008; Enge et al., 2011). However, T941G (rs6323), another important functional polymorphism in exon 8, has been related to high (941G) and low (941T) *MAOA* enzyme activities, particularly among Asians (Fan et al., 2010). The G-allele of T941G single nucleotide polymorphism is associated with elevated *MAOA* enzyme activity, which results in increased amine degradation and decreased availability of neurotransmitters, such as serotonin, dopamine, and norepinephrine. By contrast, the T-allele of this polymorphism, which is associated with decreased *MAOA* enzyme activity, leads to decreased amine degradation (Hotamisligil and Breakefield, 1991).

To our knowledge, no existing studies have examined the direct effects of the *MAOA* gene on EC; however, findings on conduct disorder or attention deficit hyperactivity disorder (ADHD), which are related to EC deficits, have remained inconclusive. Some studies have shown that high-activity *MAOA* alleles are related to ADHD (Domschke et al., 2005; Xu et al., 2007) and impulsive personality traits (Manuck et al., 2000) owing to dopamine deficiency caused by increased activation of the *MAOA* enzyme (Hwang et al., 2018). However, the aforementioned effect has not been confirmed in other studies. Behavioral studies suggest that low-activity *MAOA* allele carriers may be at a high risk of conduct disorder (Caspi et al., 2002; Kim-Cohen et al., 2006; Cicchetti et al., 2012). A recent study has shown that adolescents with conduct disorders who carried low-activity *MAOA* variants exhibited relatively pronounced inactivation of the precuneus during an inhibitory task (Sun et al., 2018). In addition, functional magnetic resonance imaging studies have revealed that inhibitory control is accompanied by reduced neural activation in the anterior cingulate cortex of low-activity allele carriers (Fan et al., 2003; Passamonti et al., 2006; Cerasa et al., 2008; Zohsel et al., 2015) with a reduced anterior cingulate cortex volume (Meyer-Lindenberg et al., 2006). High-activity *MAOA* allele carriers have also exhibited improved anterior cingulate cortex activation, an EC-related brain region, while completing the Attention Network Test (Fan et al., 2003). The lack of consistency across studies may be partly attributed to the failure of classical approaches adopted to examine genetic association to incorporate interactive effects with environmental factors (Moffitt et al., 2006). However, different levels of environmental exposure may moderate a genetic disposition such that the genetic effect may only become apparent among individuals exposed to one environment and not among individuals exposed to another. Accordingly, gene–environment interactions (G × E), defined as genetic effects that are contingent on environmental effects, or vice versa, can explain why some individuals carrying a vulnerable genotype develop a disorder while others remain unaffected (Manfred et al., 2012).

Despite these significant contributions illuminating that EC and related behaviors have a genetic basis (see Eisenberg et al., 2010) that can interact with the quality of the environment in predicting EC, the nature of such interactions has yet to be determined (e.g., Kochanska et al., 2010). According to the differential susceptibility perspective, the gene moderated associations between environmental influences and developmental outcomes from the position that certain individuals are not just more vulnerable to adversity because of their genetic make-up, but disproportionately responsive to positive and negative environmental experiences and exposures (Buil et al., 2015). However, previous studies guided by the diathesis-stress model have largely focused on negative environments and to a lesser extent on positive environment (Monroe and Simons, 1991; Burmeister et al., 2008). Several studies have shown that when exposed to childhood maltreatment, individuals possessing the low-activity *MAOA* allele may confer an increased risk of conduct disorder (Caspi et al., 2002; Kim-Cohen et al., 2006; Cicchetti et al., 2012). Denson et al. (2014) found low-activity allele carriers to exhibit increased dorsal anterior cingulate cortex and amygdala activation underlying anger control after being insulted. However, some studies (Sjöberg et al., 2007; Åslund et al., 2011) have reported that *MAOA* high-activity alleles confer vulnerability to stress (Widom and Brzustowicz, 2006), whereas *MAOA* low-activity variants are protective. For instance, low-activity *MAOA* genotypes have been associated with decreased mood problems, lowered severity of depression, and reduced symptoms of alcohol abuse in victims of sexual abuse (Nikulina et al., 2012). To our knowledge, most studies on gene–environment interactions have focused on negative environmental effects, such as maltreatment and sex abuse. That is, these studies did not look explicitly for the bright side of differential susceptibility. Therefore, this study was aimed to address this gap by exploring the potential interactions between *MAOA* gene and positive parenting rather than negative environments.

A possible reason for the inconsistency may be that the association between G × E and EC is moderated by gender. Previous evidence has suggested that *MAOA*–environment interactions on males and females can differ from each other. Several studies have reported that *MAOA* polymorphism moderates the association between the environment and EC-related behaviors among adolescent boys, including conduct disorder (Sun et al., 2018), delinquency (Foley et al., 2004), and aggression (Caspi et al., 2002; Wang et al., 2018). However, the interaction of *MAOA* with psychosocial risk has been found to affect the delinquent behavior of females (Sjöberg et al., 2007). Notably, research has also revealed opposite-direction interactions between *MAOA*–VNTR and maltreatment among boys and girls, suggesting that when experiencing maltreatment, boys with the short *MAOA*–VNTR genotype and girls with long *MAOA*–VNTR polymorphism conferred increased risks for delinquency (Åslund et al., 2011). Conversely, other studies identified no significant interactions between maltreatment and *MAOA*–VNTR in boys (Young et al., 2006; Prichard et al., 2008). These results indicated that gene–environment interactions may exert discrepant effects depending on sex. Thus, the present study investigated the potential gender differences in G × E as well.

In summary, although several studies have evaluated the G × E interaction effects on EC and related behaviors, no study has detected an interaction between the *MAOA* gene and parenting environment. Owing to insufficient relevant evidence, we did not propose a specific hypothesis on the specific allele that confers sensitivity to the environment. The current research aimed to study a large sample of adolescents to explore how *MAOA* T941G polymorphism interacts with parenting practices in predicting EC in adolescents and whether the effect of this interaction on boys and girls differ from each other.

## Materials and Methods

### Participants

Participants consisted of 1,531 Chinese students in Grade 8 (46.5% girls) recruited from 11 public middle schools in Jinan, Shandong Province, Eastern China. The mean age of the participants was 13.76 years (range: 12–15 years). Most of the participants (86.3%) were adolescents without siblings. The parents of most adolescents had earned a college/university education or higher (71.1% of fathers; 61.2% of mothers). The remaining parents had either a high school education (19.8% of fathers; 25.6% of mothers) or a middle school education or less (9.1% of fathers; 13.2% of mothers). The respective occupational prestige of mothers and fathers was as follows: 12.0% of mothers and 5.5% of fathers were peasants or unemployed, 22.9% of mothers and 24.1% of fathers held blue-collar positions, and 65.1% of mothers and 70.4% of fathers engaged in professional or semiprofessional occupations. Given indeterminate expression for heterozygous females, they were excluded from analyses (*N* = 333) (Pickles et al., 2013; Zhang et al., 2016).

### Procedures

Participants were asked to collect saliva samples using the Oragene DNA self-collection kit in accordance with the instructions provided by the manufacturer and as instructed in detail by trained researchers in their classroom. We obtained approval from the local ethics committee; in addition, informed assent and consent to participate in the study were obtained from the adolescents and their parents. The participants completed self-reported temperament and parenting style surveys during class hours or immediately after school. Research assistants were present to address the questions of the participants as needed and to ensure confidential and independent responses.

### Measurements

#### Effortful Control

The participants completed a self-reported version of the short form of the Early Adolescent Temperament Questionnaire–Revised (EATQ–R) EC scale, consisting of 16 items (Capaldi and Rothbart, 1992; Ellis and Rothbart, 2001). The activation control dimension includes five items assessing the ability of the respondents to perform an action when a strong tendency to avoid it exists. The attention dimension includes six items assessing the capacity to focus and shift attention when desired. The inhibitory control dimension includes five items assessing the capacity to plan and suppress inappropriate responses. Items were rated on a 5-point scale ranging from 1 (almost always untrue) to 5 (almost always true), with higher scores demonstrating higher EC. The measure exhibited adequate internal consistency (α = 0.81). We conduct confirmatory factor analysis (CFA) in software Mplus (Muthén and Muthén, 2012) to evaluate the internal structure of the instrument, applying full information maximum likelihood estimation to address missing data. The factor variance was set to 1 for all models to allow for estimation of item loadings (rather than setting an item loading to 1); item loadings were standardized with respect to latent variable variance (i.e., STD standardized). Preliminary CFA was run to verify the acceptability of EC measurements; the model fit statistics were as follows: CFI = 0.90, RMSEA = 0.08, and TLI = 0.90 (Hu and Bentler, 1999).

#### Parenting Practices

The Parenting Style Index (Steinberg et al., 1992) was completed by the participants. The measure included three subscales of acceptance/involvement, psychological autonomy granting, and strictness/supervision. The acceptance/involvement subscale assesses the extent to which an adolescent perceives his/her parents as loving, responsive, and involved (nine items, α = 0.86). The psychological autonomy granting subscale measures the extent to which parents apply non-coercive, democratic discipline and encourage an adolescent to express individuality within family settings (nine items, α = 0.65). The strictness/supervision subscale measures parental monitoring and adolescent supervision (eight items, α = 0.72). Some items are scored on a 7-point scale while others are on a 3- or 4-point scale. The *z*-score of items was calculated to obtain the mean score of each dimension. Typological and dimensional approaches to parenting style have been applied in previous research (Steinberg et al., 1992, 1994). With the goal of our study considered, specific parenting practices or dimensions (e.g., acceptance or strictness) were measured to obtain a perspective on the overall parenting environment (Darling and Steinberg, 1993).

#### Genotyping

DNA was extracted from saliva by using the Oragene DNA self-collection kit (Genotech Inc, Kanata, ON, Canada). Genotyping of *MAOA* T941G polymorphism was conducted with matrix-assisted laser desorption ionization time-of-flight mass spectrometry by using the primers *MAOA*-F 5′ACGTTGGATGTGCACTTAAATGACAGTCCC-3′ and *MA OA*-R 5′-ACGTTGGATGGATTCACTTCAGACCAGAGC-3′.

### Data Analyses

All analyses were performed using SPSS Version 16.0 (SPSS Inc., Chicago, IL, United States). Separate analyses were performed for boys and girls because of the X-chromosomal location of the *MAOA* gene. As previously indicated, 333 heterozygous females were excluded from analysis. In this study, a genotype was dummy-coded into 0 = T allele (i.e., T in boys and TT in girls) versus 1 = G allele (i.e., G in boys and GG in girls).

In accordance with the purpose of this study, hierarchical regression analyses were conducted to evaluate the effects of T941G polymorphism, parenting practices, and the interaction of gene and parenting practices on EC. To screen for multicollinearity between independent variables and their interactions in the regression model, the orthogonalized method (Burrill, 2003) using standard regression procedures was applied to boys and girls. Significant gene–environment interactions were further investigated using simple slope analyses. In order to exploring gene-environment interactions, we conducted regression models separated by parenting practices and sex. That is, we conducted six regression analyses. It can be possible to conduct many, separate tests, and the probability of committing a Type I error (i.e., concluding the effect exists even when it does not) for any single test of regression analysis is quite high (Sidak, 1967). Because the significance tests for different operationalization processes of the interaction effect involve numerous non-independent tests; it can be complicated to estimate the exact overall Type I error rate across multiple comparisons (see Benjamini and Yekutieli, 2001). Therefore, to test the robustness of our results, the *p*-values were corrected to control for Type I error by using the Benjamini and Hochberg (1995) procedure.

## Results

The results showed that of the 819 boys, 482 (58.9%) were G homozygotes, and 337 (41.1%) were T homozygotes; of the 712 girls, 266 (37.3%) were G/G homozygotes, 333 (46.8%) were G/T heterozygotes, and 113 (15.9%) were T/T homozygotes. Genotypic frequencies were consistent with the Hardy–Weinberg equilibrium (χ^2^ = 0.35, *df* = 1, *p* > 0.05). Table 1 presents the correlation between variables. All parenting practices showed significant positive correlation with EC; however, *MAOA* T941G polymorphism was not associated with EC in adolescents. In addition, according to the *t*-test results, higher levels of EC were reported in girls than in boys (*t*_(__1027__)_ = −2.29, *p* = 0.02). Correlation test was conducted to identify any potential gene–environment correlation (*r*GE), which implied the association between genotype and parenting practices. An association between genotype and acceptance/involvement was found in boys; specifically, those with G alleles were likely to receive more acceptance/involvement from their parents. In the case of a significant *r*GE, we performed linear regression with the genotype predicting parenting practices and saved the residuals for inclusion in the follow-up (G × E) analyses. We then conducted hierarchical regression analyses with the main effects of genotype and parenting practices (acceptance/involvement, psychological autonomy granting, and strictness/supervision) – or the residual, in the case of *r*GE – in the first step and two-way interaction terms between parenting practices (or residual) and genotype in the second step (Nederhof et al., 2012).

**TABLE 1:** Descriptive statistics and correlations among study variables.

**Variables**	**Effortful control**	***MAOA* T941G**	**SES**	**Acceptance/involvement**	**Psychological autonomy-granting**	**Strictness/supervision**
Effortful control	–	0.00	0.12*	0.39**	0.24**	0.15**
*MAOA* T941G	0.06	–	–0.04	−0.01	−0.10	0.02
SES	0.14**	0.03	–	0.09	0.10	0.16**
Acceptance/involvement	0.44**	0.10**	0.14**	–	0.35**	0.21**
Psychological autonomy-granting	0.42**	0.01	0.15**	0.14**	–	−0.05
Strictness/supervision	0.18**	0.05	0.04	0.15**	0.05	–
Mean(SD) (Boys)	3.75 (0.53)		−0.01(0.79)	3.22 (0.55)	2.75 (0.45)	2.96 (0.52)
*Mean(SD)* (Girls)	3.83 (0.52)		0.01 (0.76)	3.30 (0.48)	2.87 (0.44)	3.23 (0.44)

Numbers above the diagonal refer to girls, and numbers below the diagonal refer to boys. *p < 0.05, **p < 0.01.

As shown in Table 2, all parenting practices positively predicted EC (boys: acceptance/involvement: *b* = 0.24, *p* < 0.001; psychological autonomy granting: *b* = 0.21, *p* < 0.001; strictness/supervision: *b* = 0.09, *p* < 0.001; girls: acceptance/involvement: *b* = 0.20, *p* < 0.001; psychological autonomy granting: *b* = 0.12, *p* < 0.001; strictness/supervision: *b* = 0.07, *p* < 0.05). No significant main effect of *MAOA* T941G genotype on EC was observed among male and female adolescents. However, a two-way interaction between the *MAOA* T941G genotype and parental acceptance/involvement in the association with EC was found among boys (corrected *p* = 0.048) but not among girls (corrected *p* = 0.25). An interaction between T941G polymorphism and strictness/supervision was not present in boys (corrected *p* = 1.02) or girls (corrected *p* = 0.053). Moreover, no significant interaction effects of *MAOA* T941G and psychological autonomy granting were found in boys (corrected *p* = 0.63) or girls (corrected *p* = 0.58).

**TABLE 2:** Hierarchical linear regression analysis of the associations among *MAOA* T941G, parenting practices, and effortful control.

**Variables**		**Males**					**Females**				
		***Δ*R*^2^***	***b(SE)***	**β**	***p***	***pi***	***Δ*R*^2^***	***b(SE)***	**β**	***p***	***pi***
Step1	SES	0.02	0.09 (0.03)	0.14	0.00	**0.00**	0.01	0.08 (0.04)	0.11	0.03	0.06
Step2	*MAOA* T941G	0.19	0.05 (0.04)	0.05	0.16	0.25	0.14	0.00 (0.06)	0.00	0.97	1.01
	Acceptance/involvement		0.24 (0.02)	0.45	0.00	**0.00**		0.20 (0.03)	0.36	0.00	**0.00**
Step3	*MAOA*×acceptance/involvement	0.01	0.04 (0.02)	0.08	**0.02**	**0.048**	0.01	0.04 (0.03)	0.07	0.16	0.25
Step1	SES	0.02	0.09 (0.03)	0.14	0.00	**0.00**	0.01	0.08 (0.04)	0.11	0.03	0.06
Step2	*MAOA* T941G	0.16	0.05 (0.04)	0.05	0.18	0.25	0.05	0.03 (0.06)	0.02	0.60	0.68
	Psychological autonomy-granting		0.21 (0.02)	0.40	0.00	**0.00**		0.12 (0.03)	0.22	0.00	**0.00**
Step3	*MAOA*×Psychological autonomy-granting	0.00	0.01 (0.02)	0.02	0.50	0.63	0.00	0.02 (0.03)	0.04	0.52	0.58
Step1	SES	0.02	0.09 (0.03)	0.14	0.00	**0.00**	0.01	0.08 (0.04)	0.11	0.03	0.06
Step2	*MAOA* T941G	0.03	0.05 (0.04)	0.05	0.20	0.27	0.02	0.00 (0.06)	0.00	0.97	1.01
	Strictness/supervison		0.09 (0.02)	0.17	0.00	**0.00**		0.07 (0.03)	0.13	0.02	0.05
Step3	*MAOA*× strictness/supervision	0.00	–0.002 (0.02)	–0.003	0.94	1.02	0.01	0.07 (0.03)	0.12	0.024	0.05

*pi refers to corrected *p-*values. Significant results after B-H correction are in bold.*

**FIGURE 1:**
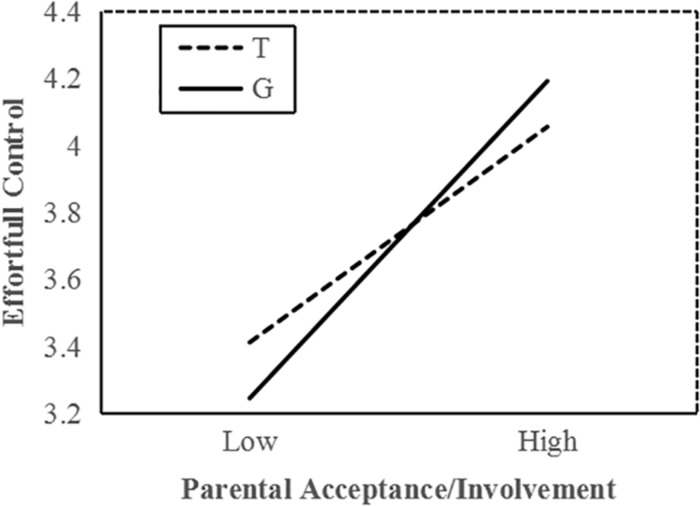
*MAOA* T941G and parental acceptance/involvement in predicting EC in boys. Simple slopes predict EC from parenting practices in different genotype groups. Solid line, G allele; dashed line, T allele.

To interpret the interaction effect between *MAOA* and acceptance/involvement, a follow-up simple slope analysis was conducted. Results indicated that among all boys, increased parental acceptance/involvement significantly predicted higher EC in G allele carriers (*b* = 0.37, *t* = 10.72, *p* < 0.001) and T allele carriers (*b* = 0.26, *t* = 6.69, *p* < 0.001); however, the interaction effects between parenting practice and two genotype carriers were significantly different from each other. In low parental acceptance, G allele carriers demonstrated lower EC than T carriers (Figure 1). Compared with T carriers, G allele carriers performed better on EC when exposed to higher parental acceptance/involvement. These statistical indices indicate that the G allele is the more sensitive genotype.

## Discussion

The present study aimed to explore the interaction effects of *MAOA* T941G polymorphism and parenting practices on EC in a large community sample of Chinese adolescents. Our study provided initial evidence for the potential interaction of the *MAOA* gene and parenting on EC in adolescents, and no significant main effect of *MAOA* T941G polymorphism on EC was found. When exposed to low parental acceptance/involvement, the adolescents with G alleles exhibited lower EC than those who with T alleles. This finding remained robust after corrections.

The results showed that higher levels of parental acceptance/involvement, psychological autonomy granting, and strictness/supervision promoted higher EC in adolescents. These findings provide additional evidence of the importance of parenting in the development of EC in adolescents, consistent with prior studies (Stacey et al., 2016; Huang et al., 2017). In contrast to parenting, *MAOA* T941G polymorphism exerted no main effect. This result further suggested that the link between a single gene and complex behavior is often too weak to be identified (Durston et al., 2005).

Notably, the results also indicated that *MAOA* T941G moderated the effect of parental acceptance/involvement on EC. Specifically, compared with the adolescents carrying T alleles, those carrying G alleles exhibited higher sensitivity to parental acceptance/involvement in predicting EC. Similarly, Larson et al. (2010) found that individuals with GG genotypes exhibited sustained eye movement to unpleasant emotional pictures (i.e., delayed recovery following negative affective stimuli), in contrast to those carrying TT genotypes. The current study suggested that the G allele was more easily affected by environment than T allele. Accordingly, we speculate that the adolescents with G genotype may be more likely to capture various emotions and information in environments (in this case, parental acceptance/involvement) by evoking emotion-related brain regions and neurophysiological over activation. By contrast, the *MAOA* T allele was presumed to be associated with less activation in the prefrontal regions, particularly the anterior cingulate cortex (Fan et al., 2003; Meyer-Lindenberg et al., 2006; Passamonti et al., 2006) and dorsal anterior cingulate cortex (Sun et al., 2018). The low activation of these brain regions may further reduce the possibility of capturing different information from the parenting environment. Therefore, these findings suggested that G alleles were the susceptible alleles, and the adolescents with G alleles were more sensitive to parental acceptance/involvement, compared with those carrying T alleles.

Another possible underlying mechanism to which G × E interaction can be attributed is that exposure to different environments alters the genetic vulnerability of adolescents by influencing the expression of a genetic factor (Johnson, 2007). That is, some genetic factors may block or suppress the influence of the environmental exposure of adolescents, leading to reduced sensitivity to different environmental effects (Sonuga-Barke et al., 2009; Sun et al., 2018). In the present study, *MAOA* T polymorphism (associated with lower monoamine oxidase function) served as a genetic factor that weakened the impact of parenting environment (in this case, parental acceptance/involvement). Such adolescents may benefit less from increased parental involvement/acceptance than G-carriers and be capable of buffering the negative effect of decreased parental involvement/acceptance, exhibiting resilient behaviors (in our study, relatively similar EC).

In this study, the G × E interaction manifested in boys but not in girls. The reason for gender differences in the interaction has yet to be determined. Gender-specific effects may be attributed to sex differences in hormonal factors that influence gene expression. Sjöberg et al. (2008) found a non-additive interaction between the *MAOA* gene and testosterone in predicting impulsive behavior, which is linked to EC. During puberty, males experience a rapid increase in testosterone, which may further alter gene–environment interactions. Moreover, the heterozygous girls were excluded from analyses given unknown patterns of X-inactivation in girls. Regardless, Carrel and Willard (2005) suggested that *MAOA* can evade X-inactivation. In the case of *MAOA* partly escaping X-inactivation, the effect of *MAOA* polymorphism on the female phenotype is more difficult to predict and may also result in non-significant G × E interaction. Therefore, explanations for gender differences in genetic susceptibility to EC remain an important direction for future research.

To the best of our knowledge, our findings provide initial evidence for the potential effects of the *MAOA* gene and parenting on EC in adolescents. Moreover, investigation of a large, community-based adolescent sample can increase the generalizability of findings. This study included participants of both sexes and identified different results of the *MAOA* × parenting interaction. Moreover, we focused on the G × E interaction in adolescence, which is a critical period for the development of EC with less attention. We also performed a Benjamini and Hochberg (1995) correction procedure to minimize the false discovery rate.

Despite these strengths, several limitations should be considered. Notably, the confounding introduced by *r*GE may reduce the reliability of the G × E interaction observed in this study. Although we sought to control for *r*GE by using statistical methods, these findings should be interpreted with caution until replicated. Second, in the current study, single informants were used to assess EC and parenting; multiple informants should be involved in subsequent research. With such a large sample, accessing EC and parenting via observational measures would be infeasible. In addition, self-report questionnaires comprise one of the most common and effective ways to assess parenting among adolescents (Chen et al., 2000; Zhang et al., 2015). Third, the current study was cross-sectional; thus, care should be taken in inferring causality between parenting and adolescent EC. Finally, given that *MAOA* polymorphism plays an important regulatory role between parenting and EC in adolescents, further consideration of the interaction including multiple genes warrants exploration.

## Conclusion

The present study provides initial evidence of the effect of the *MAOA* T941G × parenting practices interaction on EC in adolescents, emphasizing the value of testing the G × E interaction on EC and elucidating the effect of the *MAOA* gene on EC. These results also provide new insights into the interaction of genetic susceptibility with parenting in predicting EC. Our findings revealed that the G allele acts as a more susceptible factor that affords sensitivity to adolescents in parenting environments. Although further research is needed to replicate this effect and explore the potential mechanism underlying this genetic susceptibility, our study indicates that the *MAOA* T941G polymorphism × parenting practices interaction is beneficial for EC in adolescents. Our study also presents further evidence of the important effects of monoamines on EC in adolescents, contributing to the literature on the effect of the *MAOA* gene on EC.

## Data Availability Statement

The datasets for this manuscript are not publicly available due to data confidentiality. Requests to access the datasets should be directed to WZ, zhangwenxin@sdnu.edu.cn.

## Ethics Statement

The studies involving human participants were reviewed and approved by the Ethics Committee of Shandong Normal University. Written informed consent to participate in this study was provided by the participants’ legal guardian/next of kin.

## Author Contributions

BZ, YC, and WZ contributed to the conception and design of the study. BZ conducted the analysis and drafted the manuscript. YC helped in performing the statistical analysis and drafting the manuscript. LZ helped in conducting the statistical analysis. WZ conceived and coordinated the study and helped to draft the manuscript. All authors approved the final version of the manuscript for submission.

## Conflict of Interest

The authors declare that the research was conducted in the absence of any commercial or financial relationships that could be construed as a potential conflict of interest

## Abbreviations

EC: effortful control
*MAOA*: monoamine oxidase A.
